# Electrochemical Sensors Fabricated by Electrospinning Technology: An Overview

**DOI:** 10.3390/s19173676

**Published:** 2019-08-23

**Authors:** Ke Chen, Weimin Chou, Lichao Liu, Yonghui Cui, Ping Xue, Mingyin Jia

**Affiliations:** College of Mechanical and Electrical Engineering, Beijing University of Chemical Technology, Beijing 100029, China

**Keywords:** electrospinning, electrochemical sensors, glucose, hydrogen peroxide, nanomaterials

## Abstract

Nanofibers or nanofibrous membranes prepared by electrospinning possess many attractive properties, including excellent mechanical properties, high specific surface area and high porosity, making them attractive for sensor application, especially for the electrochemical sensors. Many nanomaterials are used as additives to improve the conductivity, sensitivity and selectivity of sensors. Based on the different modifiers of electrode materials, electrochemical sensors can be divided into enzyme sensors and non-enzyme sensors. In this review, we summarize the recent progress of the electrochemical sensors fabricated by electrospinning, including hydrogen peroxide (H_2_O_2_) sensors, glucose sensors and other sensors. In addition, the sensing mechanisms of various electrochemical sensors are introduced in detail. Finally, future research directions of electrochemical sensors based on electrospinning and the challenges faced by large-scale applications of electrospun electrochemical sensors are presented.

## 1. Introduction

The electrospinning technique has a history of about 80 years. It was first reported in a patent by Anton [1] in 1934 that high-voltage electrostatic fields could be used for spinning, and the production process and equipment of electrostatic spinning were described in detail. In 1964, Taylor [2] perfected the theoretical explanation of the cone formation of droplets at the spinneret nozzle during the electrospinning process. In 1995, Doshi et al. [3,4] proved the feasibility of preparing nanofibers by electrospinning, extending the electrospinning technique to the nanoscale. Since then, electrospinning technology has been widely used in scientific research and industrial fields. In recent years, more and more attention has been paid to the practical application of electrospun materials in tissue engineering [5], air and water filtration [6], solar cell electrodes [7,8], ion battery materials [9], super capacitors and sensors [10,11].

**Figure 1:**
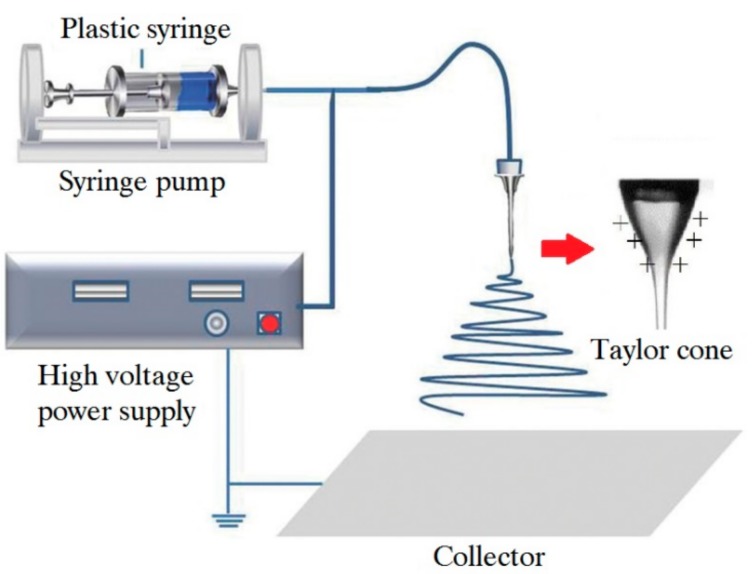
Schematic drawing of elementary setup for electrospinning.

A typical electrospinning device comprises a high-voltage power supply, a syringe pump, a spinneret with a metallic needle and a collection device (usually a metal plate or rotating shaft) [12], as shown in Figure 1. The polymer solution or polymer melt is loaded in the syringe. The viscous fluid can be driven by a micro-syringe pump to the tip of the needle. A high voltage of 10–70 kV is applied between the metallic needle, and the collection device and the conical droplet can be formed at the tip of the needle, which is known as the “Taylor cone”. When the voltage reaches a certain value, the electrostatic field force on the polymer solution or polymer melt will overcome the viscous resistance and surface tension of the fluid, and the charged jet will be ejected into the cathode. During the ejection process, the polymer solvent volatilizes or the polymer melt solidifies, thus resulting in an unstable movement or stretching. The charged jet is sharply refined and solidified into ultrafine fiber, which is randomly deposited in a spiral manner on the surface of the collection device, and then the desired fiber product is obtained.

In terms of electrospun materials, most of them are fabricated by solution electrospinning, due to its high efficiency, relative simplicity and low cost [13]. However, there are some shortcomings of solution electrospinning technology [14,15]. Common polymers, such as polyethylene (PE), polypropylene (PP) and polyamide (PA), can only be dissolved in certain solvents at high temperatures, limiting their application scope. Most polymer solvents are also harmful to the health of workers and can potentially lead to defects on the fiber surfaces [16]. Therefore, melt electrospinning was developed [17,18] in 1981 and applied to efficiently fabricate polymer fibers and composite fibers. At the beginning of the 21st century, much work was done to improve the efficiency of electrospinning technique. Dosunmu et al. [19] developed a needle-free electrospinning technique to fabricate nylon 6 nanofibers in 2006, which was 250 times more efficient than the single spinneret mehtod. Yang et al., of Beijing University of Chemical Technology, studied and developed melt differential electrospinning technology and multi-nozzle parallel connection technology, which solved the problem of the micro flow of polymer melt with high viscosity and successfully achieved large-scale preparation of nanoscale electrospun fibers [20].

The material prepared by electrospinning technology had exceptionally long length, large specific surface area, and high porosity, which can greatly improve its sensitivity and response time [21,22,23,24], making it an excellent alternative for applications in sensors, such as optical chemical sensors [25], humidity sensors [26,27], CO gas sensors [28] and electrochemical sensors [29,30,31,32]. Compared with other sensors, the electrochemical sensors fabricated by electrospinning have the advantages of high sensitivity and short response time, but have the deficiency of a short service life. More and more researchers have worked on this field in the past two decades.

The electrochemical sensor, first proposed by Clark [33] in 1962, is a device that captures the reaction between the analyte and the sensitive receptor and then expresses the degree of the reaction by mean of the electrical signal. By analyzing the magnitude of the electrical signal, we can obtain information about the solubility or content of the substance being analyzed. Taking the classical analyte H_2_O_2_ as an example, the comparison of different detection techniques is shown in Table 1. Electrochemical sensors have the advantages of high selectivity and wide detection range and have been widely used in clinical diagnosis, chemical analysis and food detection [34,35,36,37].

There are two kinds of fabrication technology for preparing electrochemical sensors based on electrospinning: one is the pretreatment method; the other is the post-treatment method. The pretreatment method is a method of introducing enzymes or nano-additive materials into the spinning solution matrix before the electrospinning process. The post-processing method is a method of treating nanofibers after the electrospinning process. Nano-additives can be loaded on electrospun nanofibers by the methods of self-assembly or hydrothermal synthesis. Electrospun nanofibers can also be carbonized to change the morphology of nanofibers to achieve high surface area and excellent electrochemical properties.

**Figure 2:**
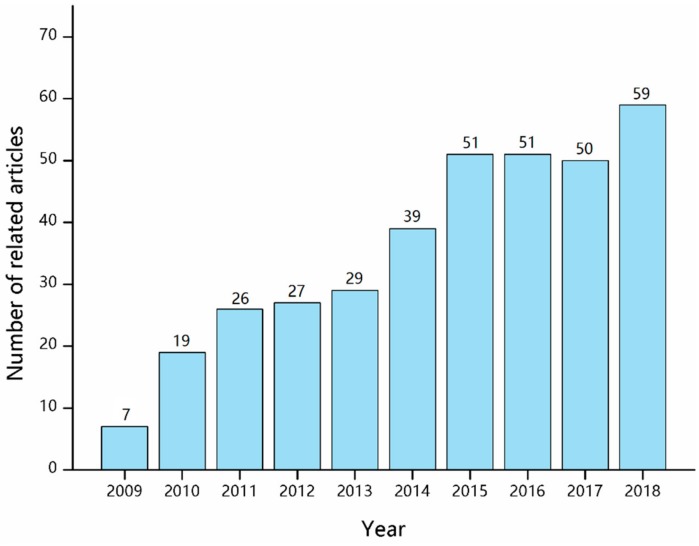
The numbers of related articles published in the last decade (data collected from Web of Knowledge).

Many researchers have reviewed the fabrication and applications of nanofibers via electrospinning [13,15,43], but some important aspects on the electrochemical sensors through electrospinning technology have not attracted enough attention. A survey of publications related to “electrochemical sensor” and “electrospinning” was performed based on Web of Knowledge, and the results are presented in Figure 2. Over the past decade, more than 400 related articles have been published. Electrochemical sensors fabricated by electrospinning are still at an early but promising stage. In this review, the sensing mechanisms of electrochemical sensors are described, and various electrochemical sensors based on the electrospinning technique are introduced in detail. Moreover, personal perspectives on future research trends and challenges of large-scale applications of the electrospun electrochemical sensors are mentioned.

## 2. Sensing Mechanism of the Electrochemical Sensor

**Figure 3:**
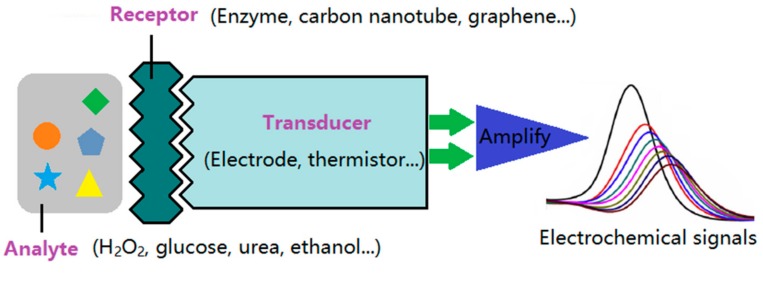
Schematic diagram of the electrochemical sensor.

The electrochemical sensor is a conversion element with an electrode as a sensor. Material with specific function on the electrode is modified as a sensitive component [44]. Figure 3 shows the schematic diagram of the electrochemical sensor. The working principle is as follows: firstly, a certain external voltage is applied to the electrode, then material with a specific electrochemical activity reacts in a redox reaction around the electrode, which generates charge transfer and further forms current. The current will be transmitted through the conduction system of the electrodes to the signal analysis system for amplification.

An excellent electrochemical sensor should have high selectivity, a low detection limit, and a short response time. The sensor system consists of two parts, namely, the receptor and the signal transducer. Each part is indispensable to the whole sensor system, and helps to promote its ultimate performance and broaden its application fields. The performance of the receptor, i.e., the detection performance of the sensor, depends mainly on the electrocatalytic activity, biocompatibility and stability of the nano-additives in the system, while the signal transduction performance mainly depends on the conductivity and distribution behavior of the nano-additives, and the electrical conductivity of the electrospun fibers.

Generally speaking, there are three kinds of electrochemical sensors: amperometric sensors, potentiometric sensors, and voltammetric sensors [45]. Among them, the amperometric electrochemical sensor is the most widely used sensor because it has characteristics of high sensitivity and good detection linearity, and is not easily affected by accidental factors in the detecting process [46,47]. When a certain applied voltage is applied, the electroactive substance produces a redox reaction near the surface of the electrode of the electrochemical sensor, leading to current generation. According to the different modifiers of electrode materials, electrochemical sensors can be classified into classical enzyme sensors and non-enzyme sensors.

## 3. Enzyme Electrochemical Sensors

Enzymes are a kind of protein with catalytic activity. They are a commonly used bio-recognition molecule in biocatalytic reaction, including various dehydrogenases and oxidases. Enzymes can catalyze innumerable complex chemical reactions of metabolism in the human body. Compared with other catalysts of various types, enzymatic catalysts have the following distinct characteristics: (a) one type of enzyme molecule can only catalyze a certain iron reaction; (b) the enzyme has a high catalytic efficiency, and each enzyme molecule can convert over 1000 substrate molecules per minute; (c) the enzyme has mild catalytic conditions and can be catalyzed at normal temperature and pressure, but is easily deactivated under high temperature, acid or alkali conditions. Because of its high sensitivity, selectivity and catalytic efficiency [48], the enzyme is usually immobilized on the electrode surface of electrochemical sensors for sensing. For the preparation of electrochemical sensors, the immobilization of enzymes is a very complex procedure and has a significant influence on the performance of the sensors [49,50].

### 3.1. Glucose Sensors

Electrospun nanofibers have characteristics of high porosity, large specific surface area and good interconnection, proving their excellent ability to immobilize enzymes [51]. Many researchers have worked on the fabrication of enzyme electrochemical sensors by electrospinning. For example, Sapountzi et al. [34] fabricated an electrochemical biosensor for detecting glucose by directly electrospinning blends of poly(vinyl alcohol) (PVA), poly(ethyleneimine) (PEI) and glucose oxidase (GOD). The electrospun PVA/PEI/GOD nanofibers were used to modify the electrode of the sensor. The prepared electrochemical biosensor of impedimetry had a linear range of 0.01–0.2 mM and a very low limit of detection (0.9 μM).

**Figure 4:**
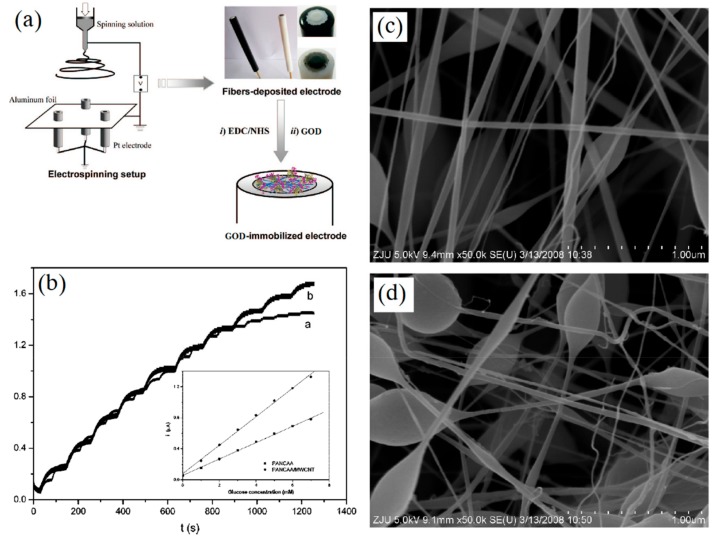
Electrospun MWCNT filled PANCAA enzyme electrochemical sensor for glucose sensing: (**a**) Schematic presentation of electrospinning for GOD electrode; (**b**) amperometric curves with various glucose concentrations; (**c**) SEM images of electrospun PANCAA NFMs; (**d**) SEM images of electrospun MWCNT filled NFMs. (reproduced with permission from reference [54]).

Carbon nanomaterials can improve the performance and lifetime of glucose sensors [52,53]. Wang et al. [54] utilized multiwalled carbon nanotubes (MWCNT) to enhance the activity of GOD for glucose sensing. The poly(acrylonitrile-co-acrylic acid) (PANCAA) nanofibrous membranes (NFMs) filled with MWCNT were electrospun and deposited on Pt electrodes, as shown in Figure 4. The experimental results indicated that MWCNT did indeed enhance the sensitivity of immobilized GOD, and that the sensor could be used more than 6 times with little decrease in current. Furthermore, the nitrogen-doped carbon nanotube (NCNT) was also an ideal carbon nanomaterial to immobilize and maintain the high electrochemical activity of enzymes due to its biocompatibility and multiple active sites. A three-dimensional (3D) NCNT/carbon nanofibers (NCNT/CNFs) composite film was prepared by Zhang et al. [55]. The NCNT/CNFs composite was directly dropped onto the electrode surface and the loading of immobilized GOD can reach 3.2 × 10^−9^ mol/cm^2^, which indicated that the prepared glucose sensor had an excellent sensitivity of 24.8 mM^−1^·cm^−2^. The 3D structure and biocompatibility of NCNT/CNFs composite provided the sensor with a long lifetime up to 4 weeks when kept at 4 °C, and the sensor still remained 92% original activity. Liu et al. [56] prepared N-doped carbon nanofibers (NCNFs) by electrospinning PAN nanofibers and subsequent carbonization. GOD and NCNFs were utilized to modify GCE of a biosensor for detecting glucose. The as-prepared glucose sensor exhibited high sensitivity and stability owing to the unique free-standing structure of NCNFs.

NFM prepared by electrospinning is also a promising carrier for the immobilization of enzymes [57]. NFM has the characteristics of high surface availability, improved storage stability, and low barrier to diffusion [58]. Arecchi et al. [59] electrospun nylon-6 NFMs to load GOD for glucose sensing. Compared with nylon film, the NFM displayed superior GOD loading and the immobilization of enzymes had no effect on their activity.

### 3.2. Other Enzyme Sensors

**Figure 5:**
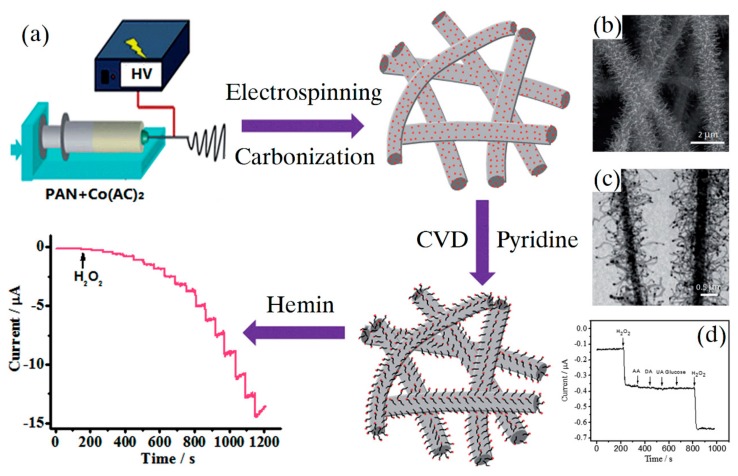
(**a**) Schematic presentation of the fabrication of NCNT/CNFs composite; (**b**) SEM images of the NCNT/CNF composite; (**c**) TEM images of the NCNT/CNF composite; (**d**) selectivity of the NCNT/CNF composite. (Reproduced with permission from [60]).

In addition to glucose, there were also some other detection targets detected by enzyme electrochemical sensors fabricated by electrospinning. Zhang et al. [60] developed a NCNT/CNF composite via electrospinning and chemical vapor deposition for sensitive detecting hydrogen peroxide (H_2_O_2_), as shown in Figure 5. Owing to high nitrogen doping and 3D structure of the NCNT, high hemin loading, low detection limit (0.03 μM) and wide linear range (0.08–137.2 mM) were achieved. Furthermore, the prepared sensor exhibited a long lifetime up to 4 weeks, stored at 4 °C and remained 93.2% original activity. The NCNT/CNF composite can be a wonderful platform for fabricating highly sensitive and stable enzyme sensors.

Li et al. [61] fabricated a catechol electrochemical sensor by dropping the mixed solution of laccase, Nafion and CNFs on the glass carbon electrode (GCE). The CNFs were prepared by electrospinning and subsequent carbonization process. The prepared catechol sensor had a wonderful electrocatalytic activity, wide linear range (1–1310 μM), which was superior to other laccase-based sensors. Comparison of various enzyme electrochemical sensors based on electrospinning is shown in Table 2.

However, in the actual detection process, the sensing abilities of these enzymes were easily affected by the differences of pH degree and temperature, resulting in the limited lifetime of sensors [62]. Taking GOD as an example, it exhibits great sensing ability only in the pH range from 2 to 8 and at temperatures below 44 °C, even though it possesses better stability than other enzymes [63]. To ensure the stability of enzymes, researchers are constantly developing new fabrication techniques, including electropolymerization of enzymes within a polymer [36], covalent cross-linking of enzyme at the pre-treated electrode surface [64] and sol–gel entrapment of enzyme [65].

However, there is still a long way to go for enzyme electrochemical sensors to work stably for a long period. The unstable nature of enzymes during immobilization also affects the stability and sensitivity of the electrochemical sensors. No matter which enzyme is used and the manner in which it is immobilized, enzyme electrochemical sensors cannot offer complete repeatability, making the large-scale production and application of artificial enzyme sensors very difficult. This Achilles’ heel of enzyme electrochemical sensors limits their application greatly, and has promoted the rapid development of non-enzyme electrochemical sensors.

## 4. Non-Enzyme Electrochemical Sensors

Non-enzyme electrochemical sensors are based on the reaction catalyzed by a variety of electrocatalyts, including metals (Au, Ag, Pt, etc.), metal oxides (CuO, Co_3_O_4_, etc.) and carbon materials (carbon nanofibers, carbon nanotubes, graphene quantum dots, etc.) [66]. Compared with traditional enzyme sensors, non-enzyme electrochemical sensors have overcome their dependence on enzymes, offering high stability, reproducibility and wide detection range, thus greatly expanding people’s understanding of the sensor, although the detection performance of non-enzyme electrochemical sensors is slightly lower than that of enzyme electrochemical sensors.

The appearance of nanotechnology has opened a new door for the application of nanomaterials in the electrochemistry field [67]. Nanostructured electrochemical materials were expected to solve the problems of poor selectivity and surface contamination with respect to the electrodes of non-enzyme electrochemical sensors. Nanomaterials can be used as additives to improve the sensitivity of the sensing system. The higher the electrocatalytic activity of nano-additives in non-enzyme sensors was, the better their detection performance would be. The signal transduction performance mainly depended on the electrical conductivity and distribution of nano-additives. Therefore, good conductors of electrical signals such as carbon nanofibers, carbon nanotubes, graphene, metal nanoparticles and other materials are widely used as additives in non-enzyme electrochemical sensor.

**Figure 6:**
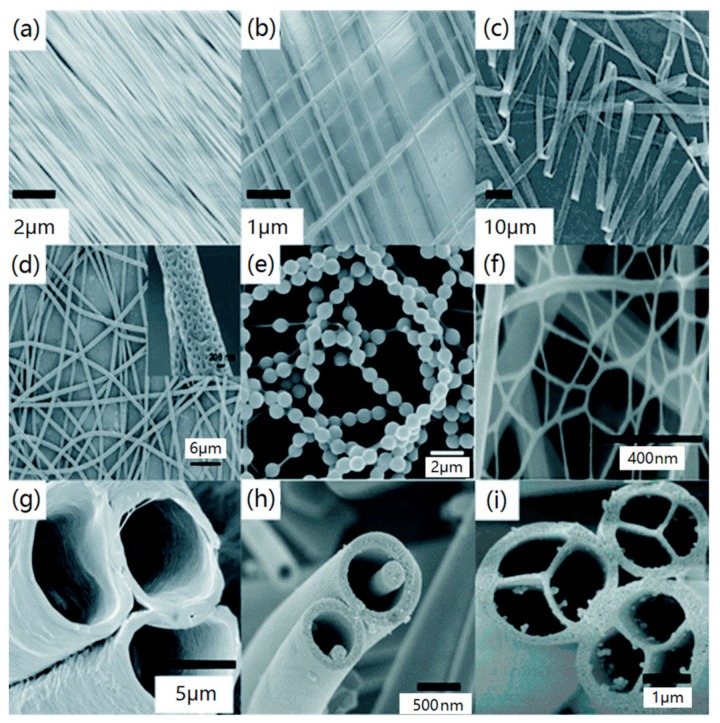
Different images of electrospun nanofibers by SEM: (**a**) unidirectional arranged; (**b**) crosswise arranged; (**c**) ribbon; (**d**) porous fibers; (**e**) necklace-like; (**f**) nanowebs; (**g**) hollow; (**h**) nanowire-in-microtube; (**i**) multichannel tubular. (Reproduced with permission from reference [22]).

Electrospinning is gaining increased interest for the purpose of fabricating non-enzyme electrochemical sensors, driven by the expectation that the surface structure of electrospun nanofibers could lead to new and exceptional effects, like controllable fiber size, structure and high chemical activity [68]. Figure 6 shows the various morphologies of electrospun polymer nanofibers. Compared with smooth fiber morphology, porous, hollow and core-shell fiber structures have larger surface area and more fixed sites, which is conducive to improving the performance of electrochemical sensors.

These characteristics have played an important role in promoting the frame function of electrospun nanofibers in electrochemical sensors. By introducing phase materials such as carbon nanomaterials, graphene quantum dots and metal nanoparticles into the spinning solution matrix, researchers can control the physical properties and electrochemical catalytic activity of the prepared nanofibers to achieve oriented loading of nanoparticles on the fibers surface, greatly improving its detection performance [69,70]. More importantly, the fiber-forming properties of these doped polymers are almost unaffected by reasonable control of nanoparticles amount.

In this section, applications of electrospinning in non-enzyme electrochemical sensors will be introduced, mainly focusing on H_2_O_2_ sensors, glucose sensors, dual-purpose sensors and some other sensors.

### 4.1. H_2_O_2_ Sensors

H_2_O_2_ is a very simple and unstable compound, which can be easily decomposed into oxygen and water. It has been widely used in various fields, such as medical disinfection, the printing and dyeing industry, the chemical industry, and environmental analysis [71,72,73]. It is also an intermediate substance in many oxidase catalytic reactions in organisms. However, if the concentration of H_2_O_2_ solution or vapor is too high during utilization, it will lead to strong corrosion and irritation to human skin, eyes or mucous membrane, harming human health. In addition, excessive H_2_O_2_ can also be harmful for cells when existing in the human body [74]. Therefore, it is important to detect the presence and concentration of H_2_O_2_ in cell metabolism [75].

**Figure 7:**
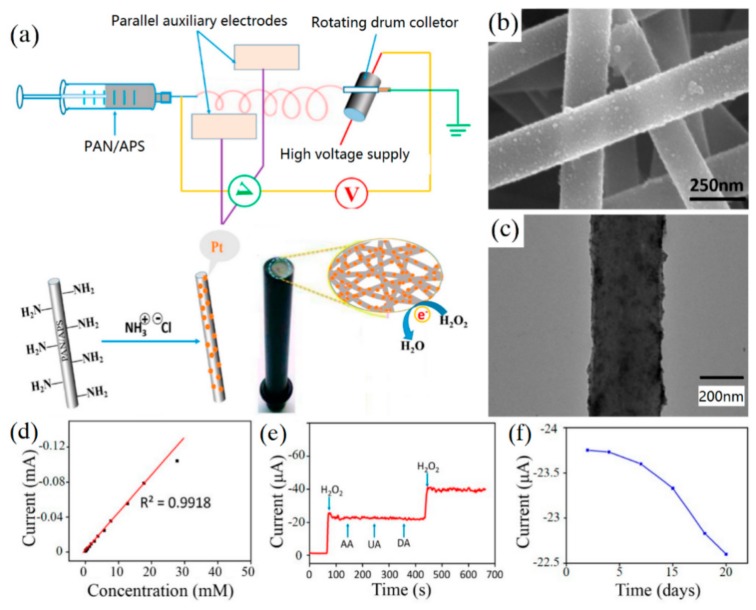
(**a**) Schematic of electrospinning apparatus for the fabrication of PAN/APS composite nanofibers; (**b**) SEM image of PAN/PtNPs composite nanofibers; (**c**) TEM images of electrospun PAN/PtNPs; (**d**) corresponding linear relationship between concentration and current; (**e**) selectivity of the sensor; (**f**) stability of the sensor. (reproduced with permission from reference [80]).

It has been reported that gold, silver, platinum, and other metal nanoparticles (MNPs) have excellent electrocatalytic activity for H_2_O_2_ [76,77,78,79]. Li et al. [80] conjugated platinum nanoparticles (PtNPs) on the electrospun NFM of polyacrylonitrile (PAN) and 3-aminopropyltriethoxysilane (APS), as shown in Figure 7. Then the prepared PAN/PtNPs nanofibrous membrane was used to modify a glass carbon electrode of the H_2_O_2_ non-enzyme electrochemical sensor. The fabricated sensor displayed a low detection limit of 1.46 μM and good selectivity towards H_2_O_2_, owing to the uniform dispersion of PtNPs. The lifetime of the sensor was up to 15 days and the current response only decreased by approximately 5% after 6 measurements. Ouyang et al. [11] used CNT and silver nanoparticles (AgNP) as additives to fabricate electrospun polyurethane/carbon nanotubes/silver nanoparticles (PU/CNT/AgNP) nanofibers. These nanofibers were directly used to modify a GCE for detecting H_2_O_2_. The synergistic effect of CNT and nano-Ag with different dimensions increased the conductivity of composite nanofibers and the prepared sensor had strong electrocatalytic activity for H_2_O_2_. The performance test showed that the linear detection range of the sensor was 0.5–30 mM, the detection limit is 18.6 μM, and the sensitivity is 160.6 μA·mM^−1^·cm^−2^.

Wang et al. [66] prepared a non-enzyme H_2_O_2_ sensor with hollow copper oxide (CuO)-modified carbon paste electrode (CPE). The hollow CuO particles were prepared by electrospinning the mixed solution of cupric acetate (Cu(Ac)_2_) and polyacrylonitrile (PAN) and subsequent carbonization. The prepared sensor had good stability and maintained 95.2% current response after 30 days.

CNF has also been widely used as a potential carrier material for electrocatalysis since its discovery [81]. Li et al. [21] fabricated porous CNF by combining electrospinning and carbonization, and then dropped PtNP on the surface of porous CNF. The prepared CNF/PtNP nanofibers were used to modify GCE of an electrochemical sensor for the highly sensitive detection of H_2_O_2_. Due to the uniform porous structure of CNF and the even coverage of PtNP, the sensor showed a wide linear response range (10 μM to 74.38 mM), low detection limit (1.9 μM), and high selectivity. Guan et al. [82] dispersed PtNi alloy nanoparticles on the surface of NCNFs by electrospinning and subsequent thermal treatment. The PtNi/NCNFs-modified electrode had the most excellent detecting performance for H_2_O_2_, when the mass ratios of Pt and Ni was 3:1. The fabricated sensor can be used to detect the content of H_2_O_2_ in milk and desirable detection result was achieved. Zhang et al. [83] electrospun a nitrogen-doped carbon nanoparticles-embedded carbon nanofibers (NCNPFs) film for detecting H_2_O_2_. Due to the free-standing structure and excellent electrocatalytic activity of NCNPFs, the sensor exhibited a wide linear range of 5.0 μm to 27 mM and sensitivity of 383.9 μA·mM^−1^·cm^−2^.

In addition, graphene (G) has been proved to be a very useful material in biosensor. Li et al. [84] prepared PVA and graphene hybrid NFM that doped with silver nanoparticles (AgNPs) by electrospinning technology. The electrospun NFM modified electrode exhibited wonderful electrocatalytic capacity for H_2_O_2_ because the high conductivity of graphene and uniform dispersion of AgNPs enhanced the electron transfer.

### 4.2. Glucose Sensors

In addition to H_2_O_2_, glucose is another significant analyte that has been widely investigated by many researchers in the areas of diabetes monitoring, food industries, and so on [43,85]. The concentration of glucose in the human body is an indicator of blood sugar concentration [86]. High blood sugar concentration can lead to obesity and diabetes, while low blood sugar concentration may result in hypoglycemia or epilepsy. Therefore, various types of glucose sensors, especially non-enzyme electrochemical glucose sensors, have been extensively studied.

Guo et al. [87] prepared CuO-loaded TiO_2_ (CuO/TiO_2_) hollow nanofiber film by a sol–gel process using the electrospun nanofiber film as the template. Then the prepared CuO/TiO_2_ film was used to modify the glassy carbon electrode of a glucose sensor. The CuO/TiO_2_ had porous fabric structure and good analytical performance. It was an excellent platform for constructing high performance glucose biosensors. Binary metal oxides have better electrical conductivity, compared to mono-metal oxides and are considered to be ideal electrode materials for high performance sensors. Liu et al. [88] developed NiCo_2_O_4_ nanoneedle-decorated electrospun CNF nanohybrids for detecting glucose. A wide linear range of 5 μm–19.175 mM and a detection limit of 1.5 μm can be achieved owing to the synergistic effects between well-dispersed NiCo_2_O_4_ nanoneedles and conductive electrospun CNF networks. Ni/CoO loaded CNF was also fabricated by combing anionic surfactant-assisted equilibrium adsorption and electrospinning to detect glucose [89]. The prepared sensor had a linear range of 0.25–600μΜ and a low detection limit (0.03 μΜ), which can retain 80% of the response after being kept in the refrigerator for 30 days.

Zhou et al. [90] prepared a non-enzyme glucose sensor based on 3D porous ZnO/CuO hierarchical nanocomposites (HNCs) by electrospinning. The electrode of the sensor was modified by the 3D porous HNCs with an optimal thickness of 13.5 μm. The sensor showed a high sensitivity of up to 3066.4 μ·Am·M^−1^·cm^−2^ and a low detection limit (0.21 μM) due to the special hierarchical heterojunction formation and the well-constructed 3D structure.

MNP/CNF hybrid nanofibers can be prepared by electrospinning metal precursor containing polymer nanofibers, followed by a thermal treatment [22]. This method provided the prepared hybrid nanofibers high electrochemical activity, owing to the uniform dispersion of MNPs within the framework of CNF and the high electrical conductivity of CNF. Li et al. [91] used this method to construct a series of MCo/CNF (M = Cu, Fe, Ni, and Mn) composites. The experimental results showed that the CuCo/CNF had the best detection performance for the human serum samples, owing the 3D structure of CuCo/CNF composites providing a large specific surface area. Most glucose sensors were used for detecting human blood sample. Detecting the glucose in saliva was another efficient and painless route for monitoring glucose in human body [92]. Xu et al. [93] developed a non-enzyme electrochemical sensor for detecting glucose. The indium-tin oxide (ITO) electrode of the sensor was modified by the electrospun CuO nanoparticles decorated polycaprolactone@polypyrrole (CuO/PCL@PPy) fibers. The sensor exhibited great selectivity and sensitivity towards glucose and was successfully utilized to determine the concentration of glucose in human saliva.

### 4.3. Dual-Purpose or Multi-Purpose Sensors

Dual-purpose or multi-purpose sensors are also in increasing demand, and can be used in clinical diagnosis, bioprocess monitoring and food industries.

**Figure 8:**
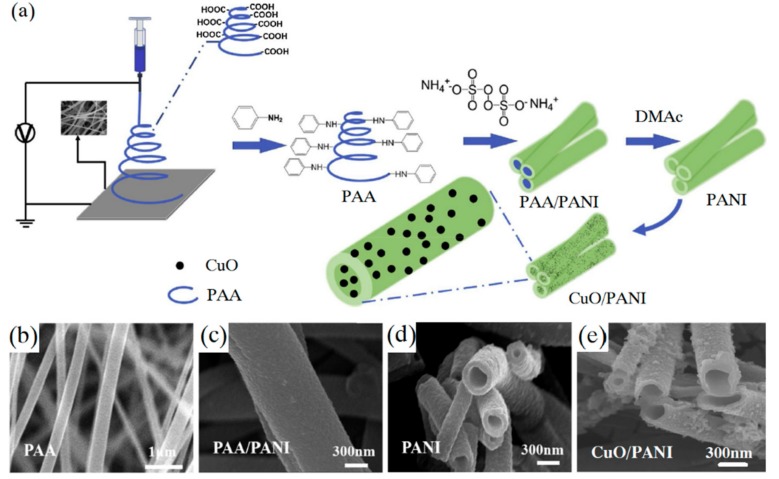
(**a**) The preparation procedure of hollow CuO/PANI hybrid nanofibers; SEM image of PAA nanofibers (**b**), PAA hybrid nanofiber covered by PANI (**c**), the hollow PANI nanofibers (**d**) and hollow PANI nanofibers doped with CuO (**e**). (Reproduced with permission from reference [62]).

Liu et al. [62] fabricated the hollow CuO/polyaniline (CuO/PANI) composite nanofibers by electrospinning. The CuO nanoparticles were doped on the nanofibers using the precipitation method. The CuO/PANI composite nanofibers exhibited great detecting performance for both H_2_O_2_ and glucose. The preparation procedure of the CuO/PANI composite nanofibers and morphologies are shown in Figure 8. It was also found that the prepared sensor exhibited high selectivity and a long lifetime of up to 10 days.

Graphene and its derivatives have the advantages of large specific surface area, good conductivity and controllable modification, and can be used as good substrates for electrochemical sensors. [94,95,96]. Zhang et al. [85] reported an electrospun PVA/graphene quantum dot (PVA/GQD) nanofibrous membrane as a dual-purpose electrochemical sensor for detecting H_2_O_2_ and glucose. The PVA/GQD nanofibrous membrane modified electrode of the sensor can directly detect H_2_O_2_, while the glucose was detected by the GOD absorbed on the nanofibrous membrane. The PVA/GQD nanofibrous membrane was also able to serve as a fluorescent sensor, since H_2_O_2_ influences the fluorescence intensity of GQDs. Zhang et al. [97] also prepared another dual-purpose electrochemical sensor based on the β-phase polyvinylidene difluoride (PVDF) nanofibrous membrane decorated with MWCNTs and PtNPs. This electrospun PVDF/MWCNT/PtNP nanofibrous membrane exhibited highly sensitive and selective detection of both H_2_O_2_ and glucose as a non-enzyme amperometric biosensor.

A dual-purpose electrochemical sensor was fabricated by Liu et al. [42] for sensing H_2_O_2_ and nitrite. The PdCo alloy nanoparticle-embedded CNF (PdCo/CNF) was prepared via electrospinning and carbonization, which was utilized to modify CPE of the sensor. Synergic effect and well-dispersed PdCo provided the sensor excellent electrocatalytic performance.

### 4.4. Other Sensors

In addition to H_2_O_2_ and glucose sensors, there are also some other electrochemical sensors based on electrospinning technology.

Zhi et al. [98,98] synthesized lanthanum strontium manganite (La_0.8_Sr_0.2_MnO_3_, LSM) nanofibers via electrospinning technology, which was used for the detection of CO at high temperatures of over 500 °C. The electrode of the sensor, which was modified by the LSM nanofibers, possessed a higher sensing ability than electrode modified by micron-sized powders. Therefore, the prepared CO sensor exhibited a wonderful electrocatalytic activity toward CO oxidation. Zhang et al. [99] prepared a sensitive non-enzyme sensor for detection of L-tryptophan, which was based on the LaCoO_3_ nanofibers prepared by electrospinning. The electrospun LaCoO_3_ nanofibers not only enhanced the specific surface area, but also promoted the electron transfer of the sensor. Li et al. [100] developed a sulfhydryl compounds electrochemical sensor with nylon-6 nanofibrous membrane (Ny-6 NFM) adsorbed with MWCNT. The prepared Ny-6-NFM/MWNT was utilized to modify the GCE of the sensor and showed great sensitivity (5.1 μA/mM) and a detection limit (15 μM). Liu et al. [101] fabricated Ni nanoparticles loaded carbon fibers (NiCFs) by electrospinning polyacrylonitrile/Ni acetylacetonate (PAN/NiAA) composite fibers and subsequent carbonization. The prepared NiCFs were used to paste on the electrode for enzyme-free sensing ethanol in wine. Compared with sensors with other transition metal-based electrodes, this sensor exhibited a superior detection performance. Guo et al. [102] reported a PdNi alloy nanoparticle/CNF (PdNi/CNF) composite prepared by electrospinning and carbonization, which was utilized as the electrode materials of electrochemical sensor for detecting sugar. The PdNi alloy nanoparticles were dispersed uniformly and embedded firmly on the surface of CNF, resulting in a very low limit of detection (7–20 nM) and wide detection linear range of 0.03–800 μM.

Ascorbic acid (AA), dopamine (DA) and uric acid (UA) were significant small biomolecules in human metabolism [103,104]. The concentrations of these substances in human body directly affect human health. For example, lack of DA can lead to schizophrenia and Parkinson’s disease. Simultaneous determination of these three substances is of great significance for the diagnosis and prevention of these diseases. Liu et al. [105] prepared a non-enzyme sensor with the function of simultaneous detection of these substances. CNFs with large amounts of edge-plane-like defective sites were electrospun and used to modify the CPE of the sensor without further pretreatment. The sensor exhibited high sensitivity and great selectivity for all these substances, owing to the high electrocatalysis of CNFs.

Pesticides such as carbendazim and atrazine are difficult to degrade and can severely affect the human health. It is essential to develop sensors for detecting these substances. Supraja et al. [106] developed a label-free ultrasensitive atrazine sensor by using electrospun SnO_2_ nanofibers, which exhibited a very low limit of detection (0.9 zM) and a detection range of 1 zM to 1 μM. The prepared sensor can detect atrazine at trace levels in spiked real-time water samples. Yang et al. [107] also synthesized nitrogen-doped hollow carbon nanofibers (N-HCNFs) by nozzle-less electrospinning for carbendazim detection. Table 3 summarizes the properties and performances of various non-enzyme electrochemical sensors based on electrospinning.

In addition, there are some electrochemical biosensors that can be used to detect biomarkers for disease diagnosis [108]. Cyclooxygenase-2 (COX-2) is an important enzyme in pain biomarkers, inflammation, and cancer cell proliferation. A biosensor for detecting COX-2 with electrospun polyaniline nanofiber was developed [109]. The reliability and sensitivity of the sensor were excellent due to the integration of electrospun polyaniline nanofibers onto interdigitated gold microelectrodes. The sensing platforms were able to detect COX-2 biomarker at concentrations as low as 0.01 pg/mL in PBS and human serum solutions, respectively. Paul et al. [110] prepared a biosensor for highly sensitive detecting carcinoma antigen-125 (CA-125), which is the most used clinical biomarker for ovarian cancer. MWCNTs embedding highly oriented ZnO nanofibers of the sensor were fabricated by electrospinning technique. The label-free sensor had a detection limit of 0.00113 UmL^−1^ concentration and a wide detection range of 0.001 UmL^−1^–1 kUmL^−1^.

## 5. Conclusions

Electrospinning is attracting increasing interest for fabricating electrochemical sensors, driven by the expectation that electrospun nanofibers have an exceptionally long length, large specific surface area, high porosity, and excellent mechanical properties. Moreover, nanomaterials, such as graphene, carbon nanotubes and metal nanoparticles, can be dispersed in the nanofibers or nanofibrous membranes as additives by combining electrospinning with other technologies. In this review, we have presented a detailed progress report on how to apply electrospinning to fabrication of enzyme and non-enzyme electrochemical sensors. The sensing mechanisms of various electrochemical sensor have also been introduced in detail.

Research into non-enzyme electrochemical sensors will be the future trend, because of their long lifetime, reproducibility and wide detection range. For the electrode of electrochemical sensors, screen-printing technology has the advantages of low manufacturing cost, easy rapid mass production and no pretreatment, which is a technique for which commercialization is easy to realize. This could not only make the electrochemical biosensor simpler, but also avoid the cross-contamination at the electrode surface. For materials that are used to modify electrodes, heteroatom doping is an effective way of modifying the surface chemical properties of electrospun composites and improving the electrocatalysis activity of sensors.

The detection performance of non-enzyme electrochemical sensors mainly depends on the distribution of nano-additives added in the electrospun nanofibers, which is also the key research emphasis in the future. It is necessary not only to understand the influence of electrospinning technology on the formation of nanofibers and the dispersion of nanoparticles, but also to understand the influence of the physical or chemical combination of these nano-additives with the nanofiber matrix on the detection performance of electrochemical sensors, in order to provide a reliable theoretical basis for the structure and performance design of the sensors. The brittleness of inorganic nanofibers prepared by electrospinning limits their application. Therefore, the development of flexible and continuous inorganic fibers is also an important subject. In recent years, electrochemical aptasensors are another promising biosensor, and can be used for detection of disease biomarkers. Metal-organic framework (MOF)-based electrochemical aptasensors have attracted increasing interest from researchers. Electrospinning is a promising method for preparing MOF nanocomposites with different systems. The diameter of composite fibers and the loading of MOFs can be adjusted by changing the concentration of polymer solution and the mass ratio of polymer to MOFs particles during electrospinning process.

Batch fabrication of electrospun electrochemical sensors is an urgent problem to be solved. Because the electrospinning speed is too slow and the output of nanofibers cannot meet the requirements of large-scale production, there are still many difficulties in large-scale industrial production of the electrospun electrochemical sensors. However, there are few studies on the efficiency improvement of electrospun nanofibers. Melt electrospinning, which is a more efficient and environmentally friendly method, may be an alternative method for fabricating electrochemical sensors. Electrospun non-enzyme electrochemical sensors will have more potential applications in material science, biomedical engineering and tissue engineering. In the future, the feasibility study on the industrialization of electrospun electrochemical sensors should be strengthened.

## Figures and Tables

**Table 1:** Comparison of performance of different techniques for detecting H_2_O_2._

Analyte	Detection Techniques	Linear Range	Detection Limit	Ref.
H_2_O_2_	Fluorophotometry	0.006–4.0 μM	81.5 pM	[38]
	High-performance liquid chromatography	0.2–100 μM	0.1 μM	[39]
	Chemiluminescence	0.14–100 μM	0.016 μM	[40]
	Spectrophotometry	0.2 × 10^–6^–14 × 10^−6^ mol·L^−1^	1.41 × 10^−6^ mol·L^−1^	[41]
	Electrochemical method	0.2 μM-23.5 mM	0.1 μM	[42]

**Table 2:** The performance of various enzyme electrochemical sensors based on electrospinning.

Analyte	Enzymes	Support Materials	Linear Range	Detection Limit	Response Time	Ref.
Glucose	GOD	PVA/PEI	0.01–0.2 mM	0.9 μM	-	[34]
	GOD	PANCAA	0–7 mM	0.557 mM	35 s	[54]
	GOD	NCNT/CNFs	0.1–12.5 mM	6 μM	40 s	[55]
	GOD	NCNFs	0.05–3 mM	0.015 mM	3 s	[56]
	GOD	Nylon-6-NFM	1–10 mM	6 μM	20–30 s	[59]
H_2_O_2_	Hemin	NCNT/CNFs	0.08–137.2 mM	0.03 μM	-	[60]
Catechol	Laccase	CNFs	1–1310 μM	0.63 μM	2 s	[61]

**Table 3:** Performances of various non-enzyme electrochemical sensors based on electrospinning.

Analyte	Electrode Materials	Detection Range	Detection Limit	Sensitivity	Ref.
H_2_O_2_	PU/CNT/AgNP	0.5–30 mM	18.6 μM	160.6 μA·mM^−1^·cm^−2^	[11]
	CNF/PtNP	0.01–74.38 mM	1.9 μM	-	[21]
	Pd-Co/CNF	0.2 μM–23.5 mM	0.1 μM	6.64 μA·mM^−1^·cm^−2^	[42]
	hollow CuO particles	0.05 μM–1 mM	0.022 μM	1746.5 μA·mM^−1^·cm^−2^	[66]
	Ag/NCNFs	0.02–20 mM	0.15 μM	142.2 μA·mM^−1^·cm^−2^	[79]
	PtNPs/PAN	5 μM–53 mM	1.46 μM	-	[80]
	Pt-Ni/NCNFs	0.5 μM–8 mM	0.0375 μM	248.5 μA·mM^−1^·cm^−2^	[82]
	NCNPFs	5 μM–27 mM	1.5 μM	383.9 μA·mM^−1^·cm^−2^	[83]
	G/AgNP	5 μM–47 mM	0.56 μM	-	[84]
Glucose	CuO/TiO_2_	0.02–19.26 mM	0.2 μM	1027.6 μA·mM^−1^·cm^−2^	[87]
	NiCo_2_O_4_/CNF	5 μM–19.175 mM	1.5 μM	1947.2 μA·mM^−1^·cm^−2^	[88]
	Ni-CoO/CNF	0.25 μM–600 μM	0.03 μM	-	[89]
	ZnO/CuO	0.47 μM–1.6 mM	0.21 μM	3066.4 μA·mM^−1^·cm^−2^	[90]
	CuCo/CNFs	0.02–11 mM	1.0 μM	507 μA·mM^−1^·cm^−2^	[91]
	CuO/PCL@PPy/ITO	2 μM–6 mM	0.8 μM	-	[93]
CO	LSM	5–500 ppm	5 ppm	-	[98]
L-tryptophane	LCPF	0.05–5 μM	0.01 μM	123.43 μA·mM^−1^·cm^−2^	[99]
Sulfhydryl	Ny-6 NFM/MWCNT	0.1–0.4 mM	15 μM	5.1 μA·mM^−1^·cm^−2^	[100]
Ethanol	NiCF	0.25–87.5 mM	0.25 mM	-	[101]
AA	CNF	0–40 μM	2 μM	-	[105]
DA	CNF	0–6 μM	0.04 μM	-	[105]
UA	CNF	0–15 μM	0.2 μM	-	[105]
Nitrite	PdCo/CNF	0.4–400 μM	0.2 μM	6.64 μA·mM^−1^·cm^−2^	[42]
Sugar	PdNi/CNF	0.03–800 μM	7–20 nM	-	[102]
Atrazine	SnO_2_	1 zM–1μM	0.9 zM	4.11 μA·mM^−1^·cm^−2^	[106]
